# Antibacterial activity and effect on gingival cells of microwave-pulsed non-thermal atmospheric pressure plasma in artificial saliva

**DOI:** 10.1038/s41598-017-08725-0

**Published:** 2017-08-21

**Authors:** Sang-Hee Seo, Ihn Han, Han Seol Lee, Jin Joo Choi, Eun Ha Choi, Kyoung-Nam Kim, Gyungsoon Park, Kwang-Mahn Kim

**Affiliations:** 1 Department and Research Institute of Dental Biomaterials and Bioengineering, Yonsei University College of Dentistry, Seoul, 03722 Korea; 2 BK21 PLUS Project, Yonsei University College of Dentistry, Seoul, 03722 Korea; 3 Plasma Bioscience Research Center, Kwangwoon University, Seoul, 01897 Korea

## Abstract

Although various oral pathogens are inactivated by non-thermal atmospheric pressure plasma (NTAPP), the *in vivo* effects of NTAPP are poorly understood. The first aim of this study was to examine the antibacterial activity of microwave-pulsed NTAPP against *Staphylococcus aureus* in artificial saliva to mimic oral environmental conditions. The second aim was to determine the influence of microwave-pulsed NTAPP on human gingival fibroblasts (HGFs). The microwave-pulsed NTAPP reduced bacterial viability (as measured by colony forming units [CFU]) to a greater extent in artificial saliva than in saline. Extending the post-treatment incubation time increased bacterial inactivation in artificial saliva compared to saline. HGFs viability was unaffected by microwave-pulsed NTAPP for bacterial inactivation. Rather, HGFs proliferation increased after a 5-min microwave-pulsed NTAPP. Less tumor necrosis factor alpha was released by microwave-pulsed NTAPP-treated HGFs stimulated with lipopolysaccharide (LPS) than by untreated, LPS-stimulated HGFs; thus, plasma appeared to suppress the inflammatory response. Our study suggests that microwave-pulsed NTAPP may have stronger *in vivo* antibacterial activity than *in vitro* activity, and that microwave-pulsed NTAPP may have the additional advantage of suppressing gingival inflammatory responses.

## Introduction

Periodontitis, an inflammatory disease affecting tooth-supporting tissues, is caused by chronic bacterial infection that destroys the gums and jaw bones. Lipopolysaccharide (LPS) and peptidoglycan from bacterial cell surfaces induce host tooth immune responses including production of pro-inflammatory cytokines and subsequent inflammatory responses^1^. After immune response initiation, host cells produce reactive oxygen species (ROS), cytokines, procoagulants, and other small molecules that amplify and sustain the inflammatory response, while causing progressive tissue destruction^2^. A variety of cytokines, including interleukin (IL)-1α, IL-1β, IL-6, IL-8, tumor necrosis factor alpha (TNF-α), CD14, and Toll-like receptors, have been detected in inflammatory gingival tissues with periodontitis^1^. TNF-α is a well-established pro-inflammatory cytokine involved in the regulation of infection, inflammation, and autoimmune phenomena^1^. Many studies have shown that some cytokines, including TNF-α, play a primary role in microbial pathogenesis^3, 4^. In severe cases of periodontitis, locally produced, pro-inflammatory cytokines can enter systemic circulation and induce an acute liver response^5^.

Safe, effective, and economic methods have been developed to cure and prevent periodontal disease by controlling bacterial infection^6^. Several antibiotics and therapies (eg, scaling, root planning, medication, surgical treatments [flap surgery], flossing, brushing, rinsing, and laser treatments) have been used to eliminate bacteria that may cause periodontal disease^6–10^. A recent study suggested that, because ROS levels are an important factor in periodontal disease pathogenesis, ROS control may play a key role in periodontal disease prevention^11^. Indeed, ROS-reducing antioxidant therapy can control bacterial infection and produce anti-inflammatory effects on periodontal tissues^11, 12^. Although there are a variety of periodontitis treatments available, the search for reliable, multidisciplinary cures is ongoing.

Non-thermal atmospheric pressure plasma (NTAPP) is being explored as a potential tool to control bacterial infections associated with periodontitis^13^. NTAPP antimicrobial activity against oral bacteria and biofilms has been demonstrated^14^. In particular, microwave-generated plasma has been used to inactivate bacteria because of its low cost, simplicity, and ease of preparation^15–17^. Needed microwave devices can be constructed at relatively low cost because electrodes are not required to generate microwave plasma^18, 19^. Short-lived reactive chemical species created by microwave plasma will destroy microorganisms by reacting with cell wall hydrocarbon bonds, then with reaction by-products such as CO, CO_2_, and OH radicals. Thus, the outer cell wall may be removed initially, and continued reactions will destroy the secondary cell wall^20^. Through this mechanism, it is possible that microwave plasma could have prolonged effects on bacterial disinfection or sterilization^21–24^. Despite these advantages, several weaknesses require technical improvement. Ozone is produced during plasma generation and plays an important role in microbe inactivation^25^; however, ozone can be inhaled during treatment. Another problem is that efficiency of plasma sterilization can vary by microbial species and environment^26^. Optimization of plasma sources is needed to make use of plasma as a general sterilization tool.

Although research to clarify the properties and mechanisms of antibacterial plasma activity is increasing^27^, substantially more evidence is needed to prove the efficacy of plasma technology in treating *in vivo* oral infection. The interaction of plasma with the oral microenvironment is poorly understood, and antibacterial activity should be tested under conditions mimicking that environment using saliva before NTAPP therapy can be used clinically. Moreover, the influence of NTAPP on surrounding tissues should be considered.

In this study, we assessed the ability of microwave-pulsed NTAPP to inactivate *Staphylococcus aureus* in artificial saliva. Secondly, we investigated the influence of microwave-pulsed NTAPP on inflammatory responses of surrounding tissue cells. Although artificial saliva does not provide a true representation of saliva, it has been used to mimic the oral environment^28^. *S*. *aureus*, a gram-positive bacteria well-known for its biofilm formation^29^, is the most commonly found staphylococcal species in the oral cavity. It is thought to be responsible for infectious endocarditis through oral-nasal trafficking^30^. Therefore, understanding the effects of plasma on *S*. *aureus* may provide information that is applicable to other oral bacteria.

## Results

### Physical and chemical properties of microwave plasma

**Figure 1:**
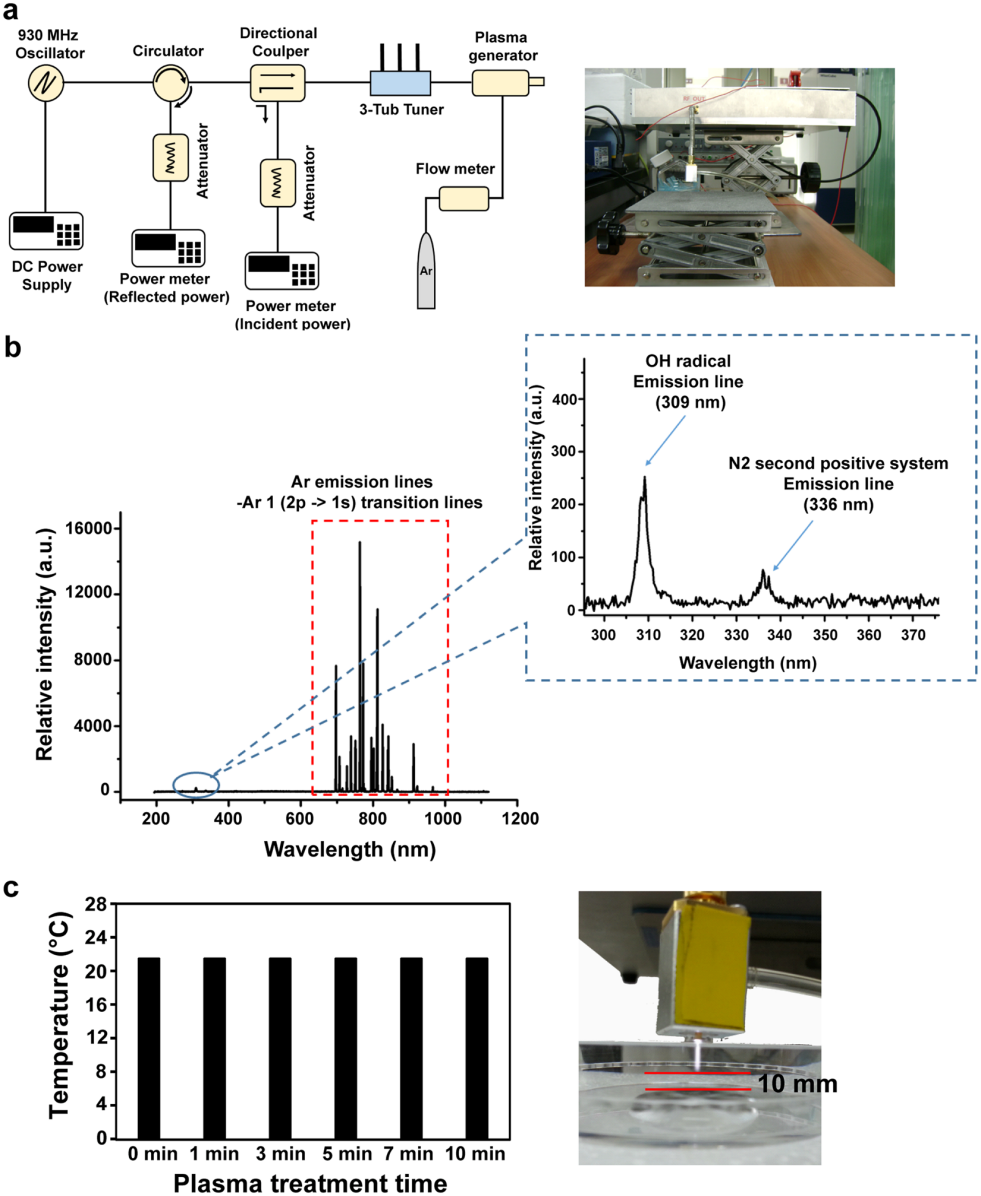
(**a**) Schematic view of microwave-pulsed non-thermal atmospheric pressure plasma (NTAPP) impedance system. (**b**) Optical emission spectra of microwave-pulsed NTAPP with Argon (Ar) gas; Ar emission lines at 650 to 950 nm; OH emission band at 306 to 309 nm; and nitrogen second positive system at 330 to 400 nm. (**c**) Temperature of deionized water treated with microwave-pulsed NTAPP for different lengths of time. The distance between the upper surface of the solution and the bottom of the plasma plume was 10 mm.

The optical emission spectrum of microwave-pulsed NTAPP (Fig. 1a) with Argon (Ar) feeding gas is shown in Fig. 1b. Ar emission lines were detected in the 650 to 950 nm wavelength range, whereas nitrogen second positive system and OH emission bands were observed at wavelengths in the 330 to 400 nm and 306 to 309 nm ranges, respectively (Fig. 1b). To optimize conditions for microwave-pulsed NTAPP, the temperature of a 1 ml water sample was monitored over different treatment times, sample-plasma plume distances, and gas flow rates. The temperature of the sample was stable at approximately 23 °C for up to 10 min when treated at 10 mm from the tip of the plasma plume with a 2 L/min gas flow (Fig. 1c). This treatment condition was used in all subsequent experiments to avoid heating effects.

### Inactivation of *Staphylococcus aureus* in artificial saliva by microwave plasma

**Figure 2:**
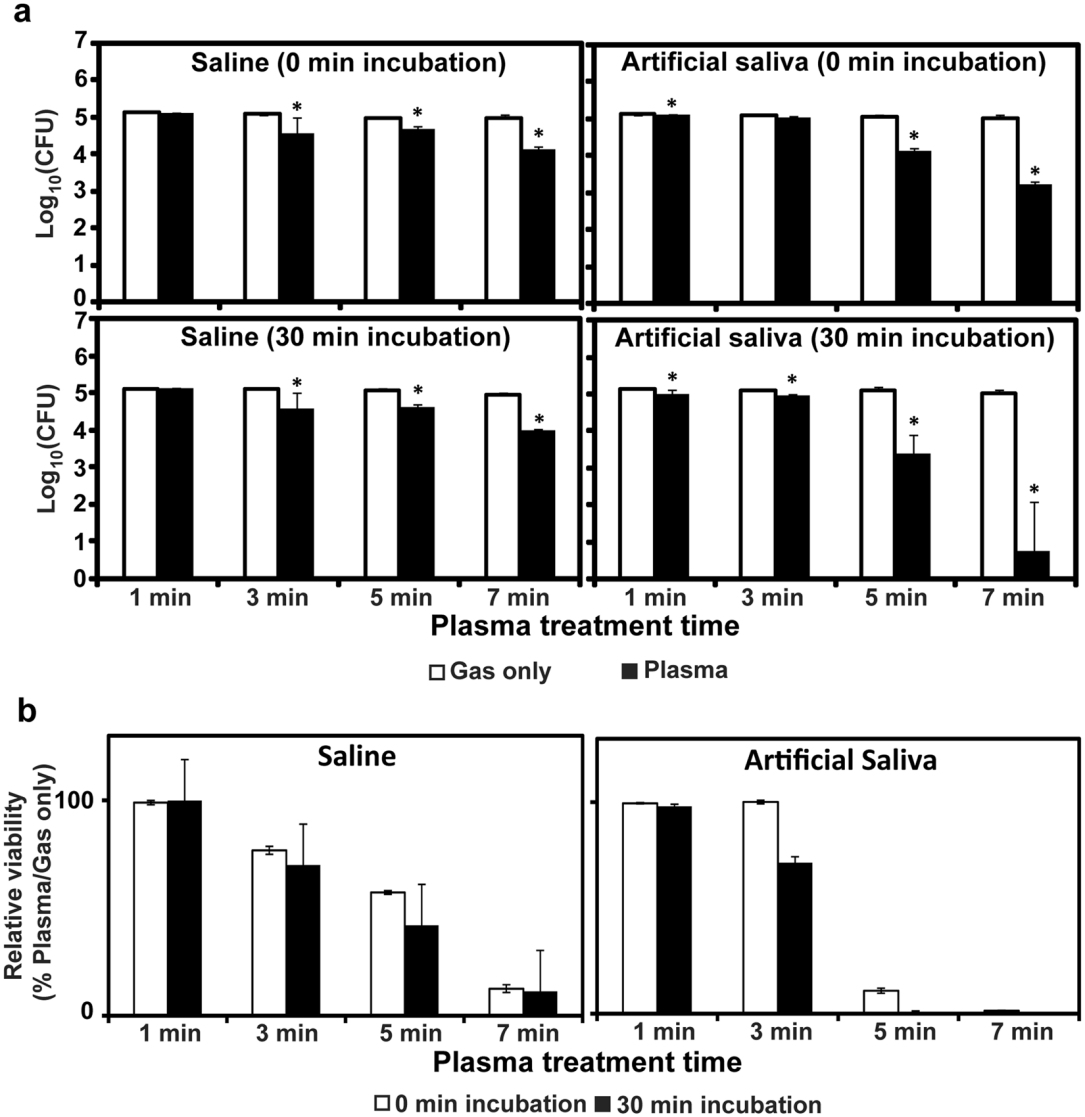
(**a**) Bacterial viability measured as colony forming units (CFU) on a log scale after plasma treatment in saline or artificial saliva, with or without 30-min incubation. (**b**) Relative viability of plasma-treated bacteria compared to gas only control treatment. Relative viability (%) = (CFU after plasma treatment/CFU after gas treatment) ×100. All values are means ± standard deviations of triplicate measurements, and all experiments were repeated three times. *P < 0.05.

Figure 2 shows the antibacterial activity of microwave-pulsed NTAPP in saline and artificial saliva. In both solutions, the numbers of viable bacteria were significantly decreased after microwave-pulsed NTAPP in a treatment time-dependent manner, with the decrease occurring more rapidly in artificial saliva compared to saline (Fig. 2a). When bacteria were incubated for 30 min after treatment, reductions in colony forming units (CFU) were greater in artificial saliva compared to saline (Fig. 2a). CFU were reduced following exposure to plasma in artificial saliva for 5 min and 7 min (*P* < 0.05 for both). Notably, we observed an almost 4-log reduction in CFU following a 7-min treatment and 30 min incubation (Fig. 2a). The relative viability of plasma-treated bacterial cells (vs gas only treatment control) was much smaller in artificial saliva than in saline for the same treatment time; the difference became more distinct after a 30-min incubation (Fig. 2b). These results indicate that our plasma is more toxic to bacteria in artificial saliva than in saline and that a longer incubation enhances this bactericidal effect.

**Figure 3:**
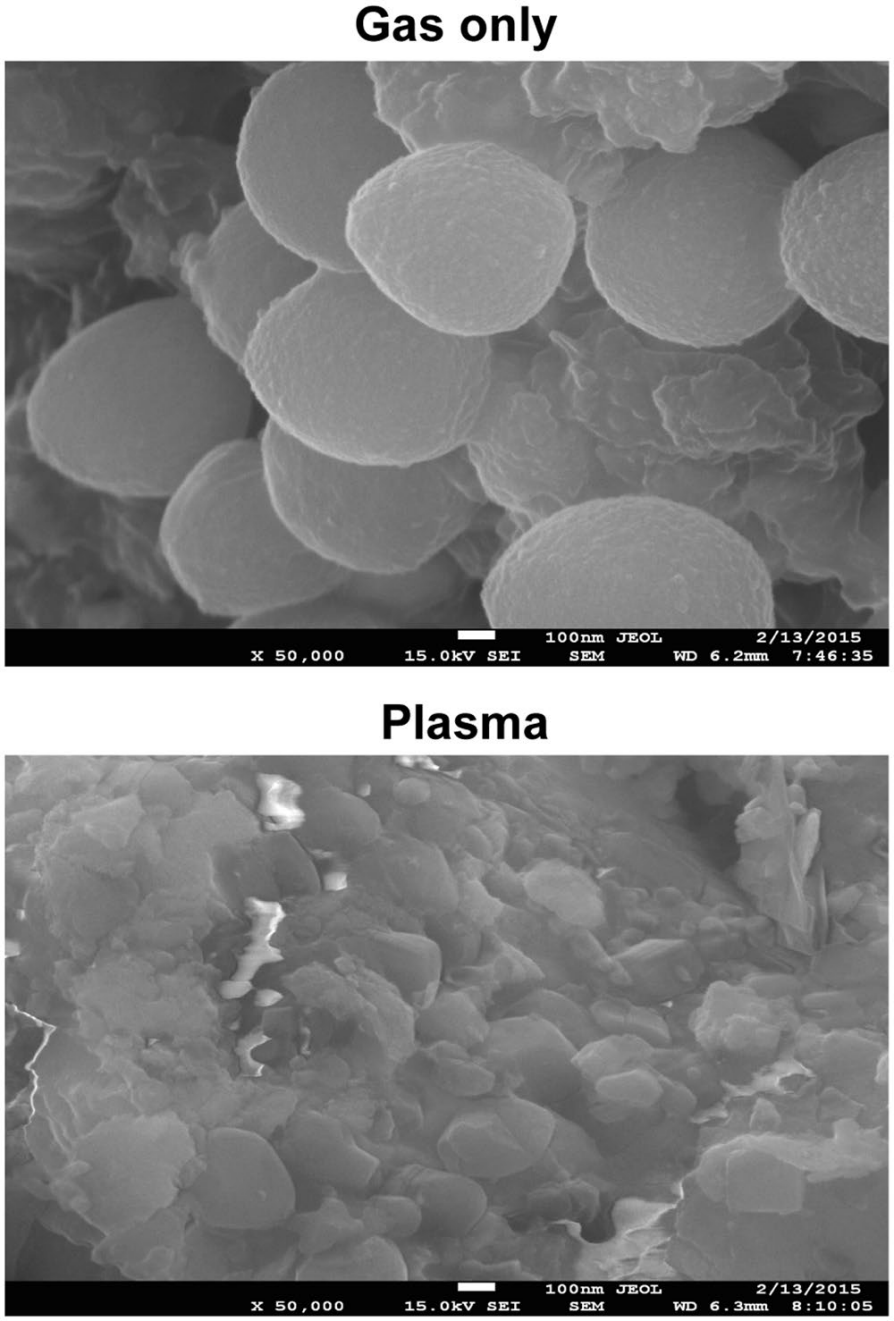
Scanning electron micrographs of *S*. *aureus* in artificial saliva after 5-min treatment with gas only (upper) or plasma (lower). Magnification, 50,000x.

Bacteria subjected to plasma treatment in artificial saliva had dramatically altered morphology (Fig. 3). Most bacterial cells treated with plasma for 5 min in artificial saliva were crushed and torn, whereas control cells treated only with gas retained their spherical shapes (Fig. 3).

### Analysis of solutions after plasma treatment

**Figure 4:**
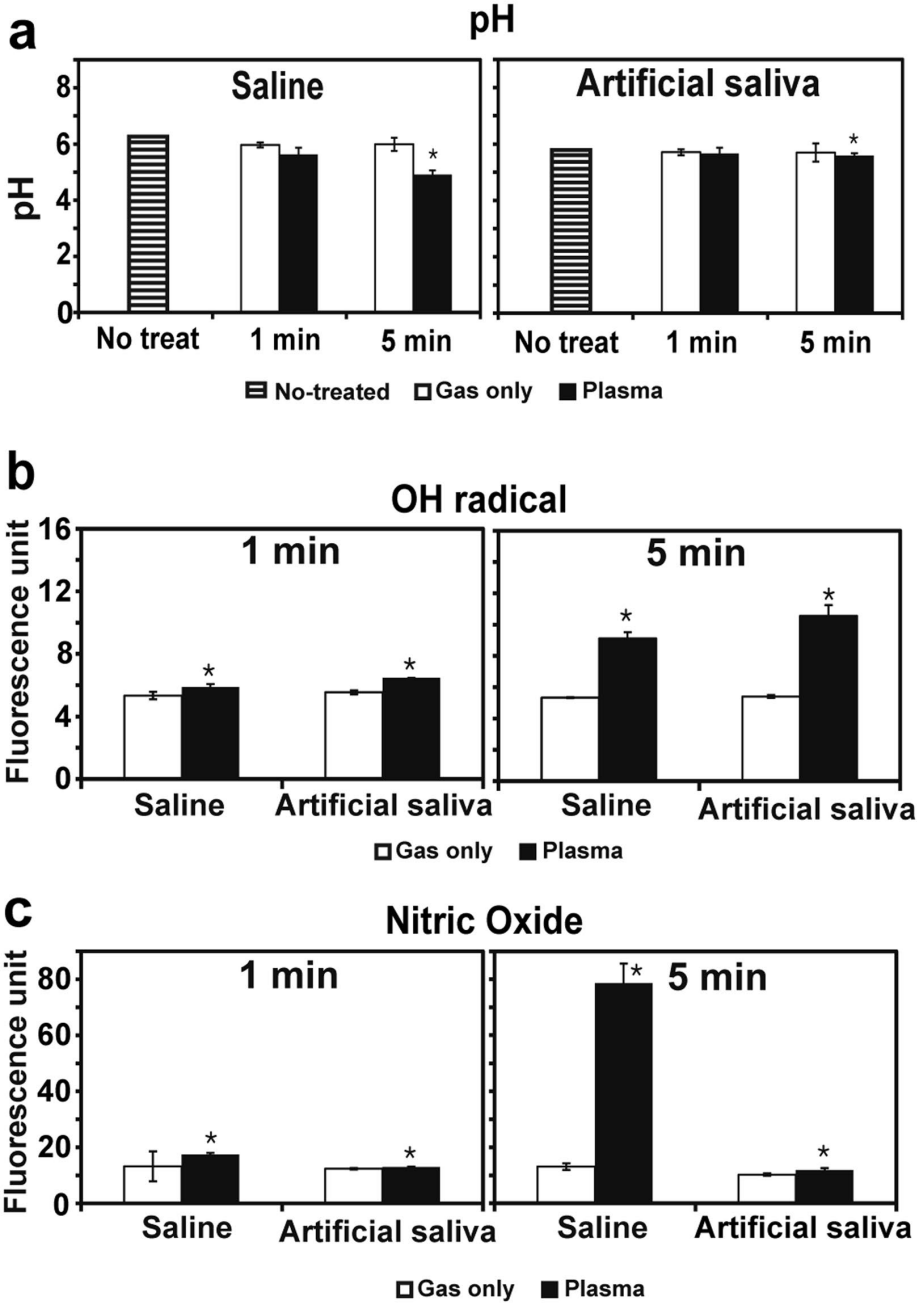
Changes in pH, OH radical level, and NO level in saline or artificial saliva after plasma treatment for 1 min or 5 min. (**a**) pH of saline and artificial saliva after gas or plasma treatment. (**b**) OH radical level in saline and artificial saliva after gas or plasma treatment. (**c**) Levels of NO species in saline and artificial saliva after gas or plasma treatment. Levels of OH radical and NO are indicated by fluorescence intensity of terephthalic acid (TA) and 4-Amino-5-Methylamino-2′,7′-Difluorofluorescein combined with OH radical and NO, respectively. All values are means ± standard deviations of nine measurements. *P < 0.05.

Plasma treatment for 5 min altered the pH of saline more than it altered the pH of artificial saliva (Fig. 4a). After a 5-min plasma treatment, the saline pH (4.91 ± 0.04) was markedly lower than the untreated saline pH (6.26 ± 0.00). In contrast, the pH of artificial saliva after 5-min plasma treatment (5.59 ± 0.03) was only slightly lower than that of untreated saliva (5.66 ± 0.03; Fig. 4a). This finding may be explained by the presence of phosphate and urea, which can act as buffers in artificial saliva. Conversely, the plasma treatment increased OH radical levels to the greatest extent in artificial saliva, to a lesser extent in saline, but not in the gas only control (Fig. 4b). The increases for artificial saliva and saline were far greater after 5-min treatment than after 1-min treatment. NO levels were increased following the 5-min plasma treatment in saline, but only negligibly in artificial saliva (Fig. 4c).

**Figure 5:**
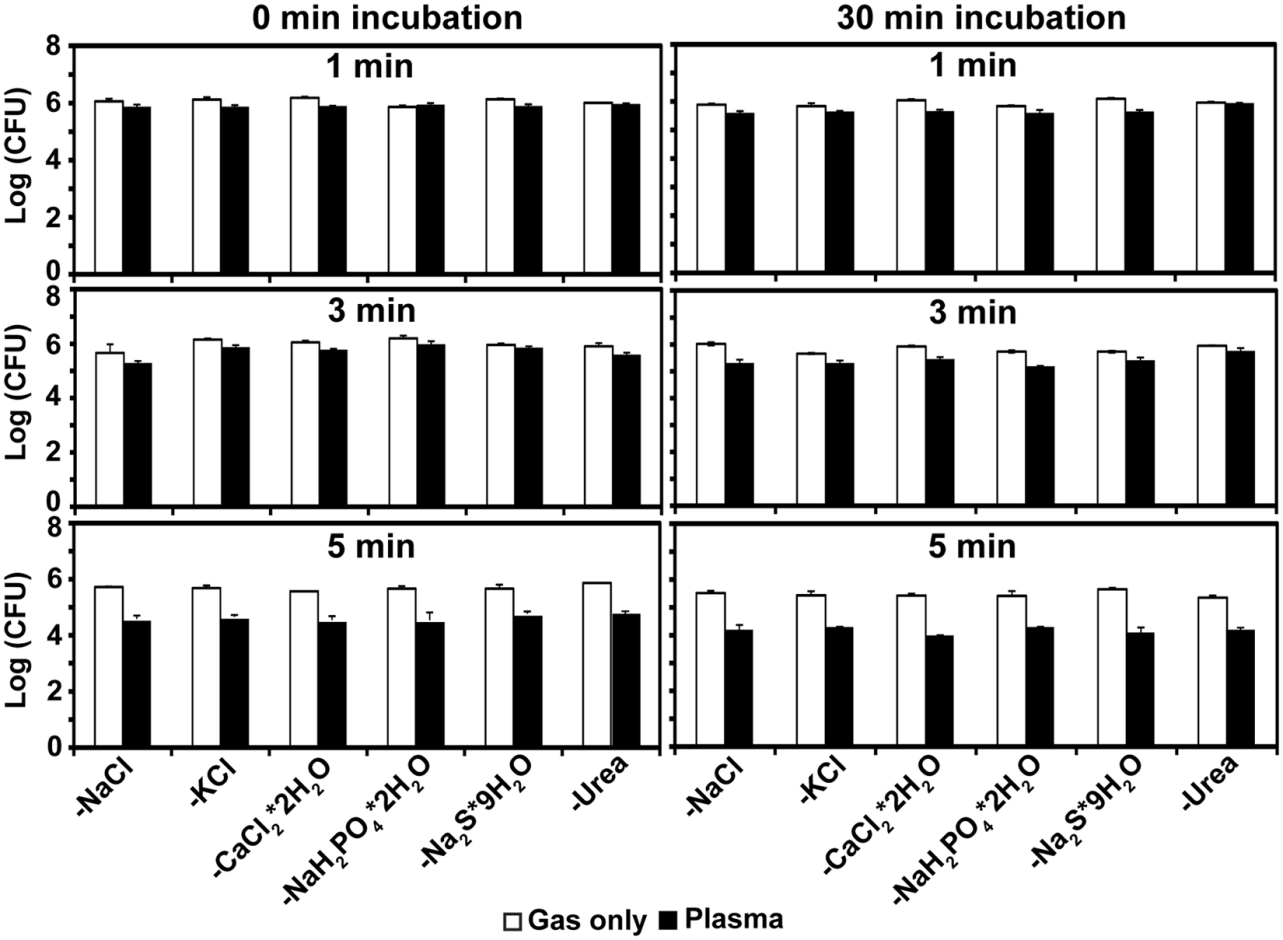
Bacterial viability, as determined by CFU on a log scale after gas or plasma treatment for 1 min, 3 min, or 5 min in saline and artificial saliva missing components. All values are means ± standard deviations of triplicate measurements.

Although OH radical levels were higher in plasma-treated artificial saliva than in saline, they were not sufficient to explain the increased antibacterial activity of plasma in artificial saliva. To investigate longer-term chemical changes that may explain the observed increased bactericidal activity in artificial saliva, we quantified bacteria viability in artificial saliva samples by individually omitting chemicals. When samples were treated with plasma for 1 min and 3 min, bacterial viability was slightly reduced in artificial saliva that lacked individual chemicals, even after 30 min of incubation (Fig. 5). There were no differences in reduction patterns by specific chemical omitted; however, the viability reduction was less pronounced in artificial saliva lacking urea (Fig. 5). Five-minute treatment reduced bacterial viability markedly when any constituent chemical was omitted (Fig. 5). These results suggest that synergistic interactions among chemicals may play a crucial role in the observed antibacterial properties of plasma.

**Figure 6:**
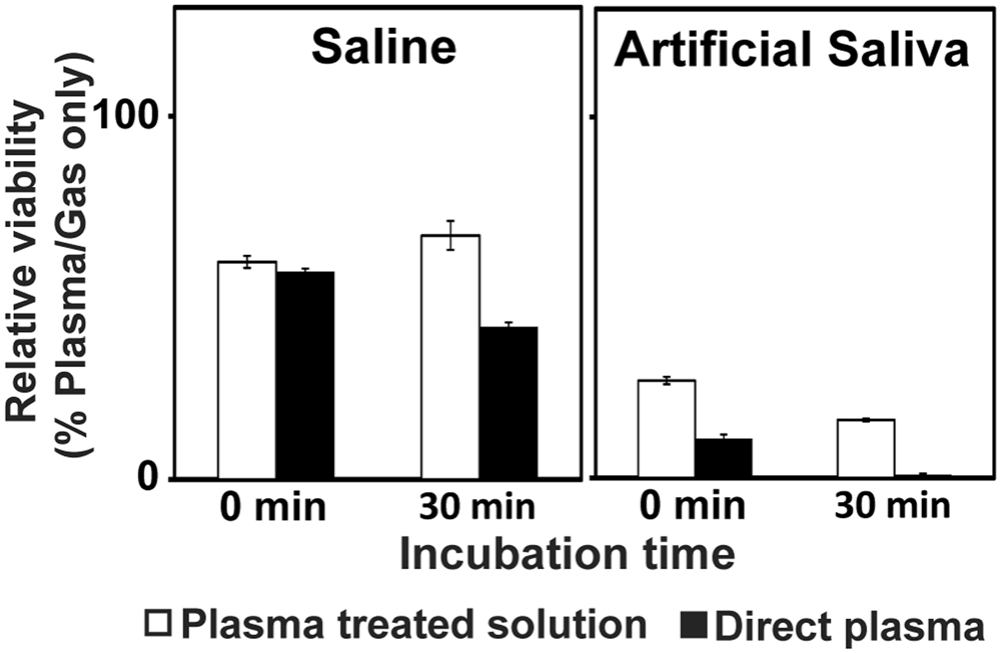
Inactivation of bacteria after treatment with direct plasma or plasma-exposed solutions. All treatments were performed for 5 min. Bacterial inactivation was indicated by relative viability of plasma-treated bacteria compared to gas only-treated bacteria. Relative viability (%) = (CFU after plasma treatment/CFU after gas treatment) ×100. All values are means ± standard deviations of triplicate measurements.

We found that continuous plasma exposure led to more bacterial inactivation (with or without a 30-min incubation) than administration of plasma-treated solutions. Bacterial viability was reduced more by direct plasma treatment in saline and artificial saliva than by treatment in saline or artificial saliva pre-exposed to plasma; this difference was amplified after a 30-min incubation (Fig. 6). Finally, bacterial viability was decreased more in artificial saliva than in saline, with or without incubation, following direct plasma treatment and following treatment with plasma pre-exposed solutions (Fig. 6).

### Viability and inflammatory responses of gingival cells after plasma treatment

**Figure 7:**
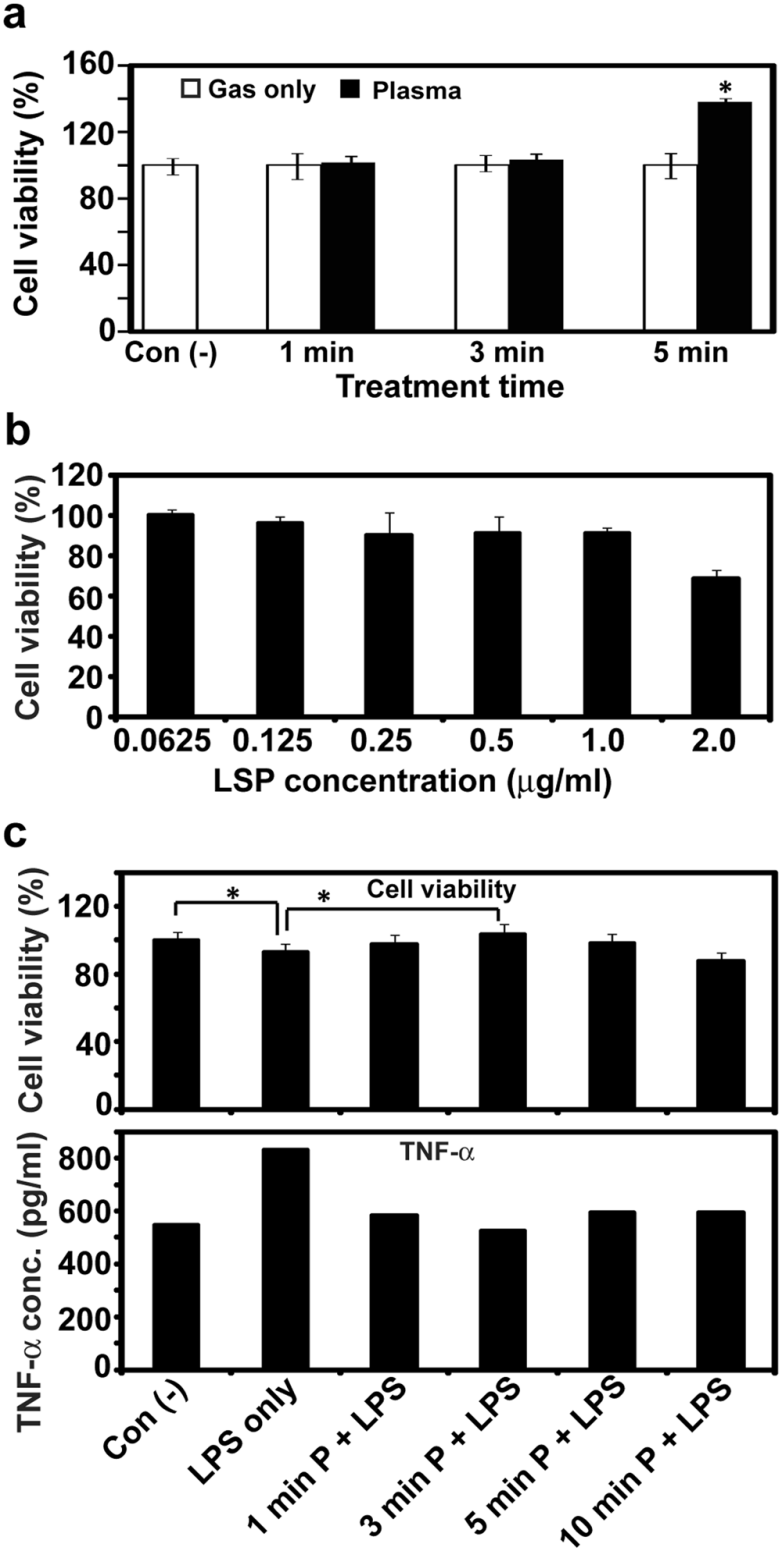
(**a**) Viability of plasma-treated human gingival fibroblasts (HGFs); *P < 0.05. All measurements were performed nine times. (**b**) Viability of HGFs stimulated with a range of lipopolysaccharide (LPS) concentrations. All measurements were performed three times. (**c**) Viability and tumor necrosis factor alpha release by HGFs treated or not with plasma (1 min, 3 min, 5 min, and 10 min) after 1.0 µg/ml LPS stimulation; *P < 0.05 vs con(−) (no LPS or plasma). All measurements were repeated eight times.

As shown in Fig. 7a, HGFs viability was not affected by 1-min or 3-min plasma treatment at the same dose described previously. Interestingly, increased cell proliferation was observed after 5 min in the plasma treatment sample compared to the gas only control treatment or no treatment.

HGFs viability did not change with LPS doses less than 1.0 µg/ml, but did decrease slightly following administration of 1.0 µg/ml or 2.0 µg/ml LPS (Fig. 7b). Thus, for subsequent cytokine experiments, a 1.0 µg/ml LPS concentration was used to induce inflammatory responses in HGFs with minimal reduction in cell viability. Levels of TNF-α released from plasma-treated cells were much lower following LPS stimulation than levels observed for untreated plasma cells, but similar to those observed in control cells that were not LPS-stimulated (Fig. 7c, bottom graph). This finding suggests that plasma treatment may be able to suppress, at least partially, the gingival inflammatory response. The TNF-α–releasing effect of LPS on HGFs was blocked by plasma treatment at 1 min, 3 min, 5 min, and 10 min. Moreover, the cell viability-reducing effect of LPS stimulation was countered effectively using plasma treatment for 1 min, 3 min (viability greater than no LPS), or 5 min (Fig. 7c, upper graph). LPS-exposed HGFs treated with plasma for 10 min, however, had reduced cell viability relative to control cells not exposed to LPS (Fig. 7c upper graph).

## Discussion

Our study results have several important implications for the management of periodontal disease using plasma *in vivo*. First, microwave plasma may be more effective in a real oral environment including saliva than under experimental conditions. Although artificial saliva does not provide a true replication of the oral environment, it did inactivate microbes at a rate that was orders of magnitude greater than the rate in saline. Until now, the effect of plasma has not been studied extensively with real saliva, but it has been used occasionally to test for corrosivity of plasma to treated titanium surfaces^28^. Because real saliva is a complex system, our results using artificial saliva may be too preliminary to apply to the oral environment. However, these results do provide some understanding of the antibacterial activity of plasma *in vivo*. We will further investigate antibacterial activity of plasma using real saliva or artificial saliva with added factors from real saliva.

Our finding that post-plasma treatment incubation amplifies bactericidal effects suggests that similar, longer-term effects may also occur *in vivo*. Although plasma effects have been reported as short-lived in previous studies^31, 32^, we found CFU reductions for at least 30 min after plasma treatment. This result may indicate that long-life active species are produced by plasma treatment in artificial saliva or saline during the incubation time. Longer-lasting sterilization efficacy has also been observed in plasma-treated solutions^33–35^. Based on our results, it may be inferred that the bactericidal effect of the oral environment can last for a while after plasma treatment. Finally, microwave plasma may dually affect pathogenic bacteria and gingival tissues through bacterial inactivation and inflammatory response suppression (as evidenced by TNF-α release), while preserving gingival cell viability. We observed that the same dose of microwave plasma that generated antibacterial activity also suppressed cytokine production in response to LPS stimulation. Furthermore, both effects occurred without adverse effects to gingival cell viability up to 24 h. These findings are noteworthy given that prior studies have demonstrated pro-apoptotic effects of plasma on mammalian cells^36–38^, and the findings support the notion that plasma therapy may be useful to control oral diseases complicated by bacterial infection.

The present results provide several clues regarding the mechanisms of action of microwave-pulsed NTAPP. Temperature and NO level did not appear to be the main factors responsible for the greater bactericidal effect of plasma in artificial saliva. According to our principal component analysis, treatment time, pH, and OH radical level affected bacterial viability in artificial saliva, but NO level was not correlated with bacterial viability (Supplementary Table S1). These findings indicate that additional factors, such as chemical alteration, may be responsible for the greater bacterial inactivation in artificial saliva.

Because several chemicals are present in artificial saliva, chemical alteration or generation of long-life species can be expected during interaction with plasma. Chemical analysis of artificial saliva treated with plasma should be performed in future studies. In this study, we found that removal of individual chemicals from the artificial saliva did not affect plasma-induced bacterial inactivation. Although removal of urea seemed to diminish the short-term bactericidal effects of plasma, the effect was trivial. Thus, synergistic chemical effects from interactions between plasma and multiple individual chemicals may play important roles in generating the bactericidal effect of plasma in artificial saliva. We did not identify any chemical composition changes in the artificial saliva that resulted from the plasma treatment, but we will explore this topic in our future investigation.

Based on our results, reactive species clearly play a crucial role in bacterial killing, although NO did not appear to contribute to bacterial inactivation in artificial saliva. NO may have benefitted HGFs viability and reduced inflammatory signaling, as it is known to do^39, 40^, especially in periodontitis^41^. Relatively greater OH radical levels in artificial saliva than in saline after plasma treatment may explain, in part, the enhanced bactericidal efficacy of plasma in artificial saliva.

Lastly, greater bacterial inactivation with direct plasma exposure in artificial saliva (rather than in plasma-pretreated artificial saliva) indicates that chemical alteration itself cannot sufficiently explain the bactericidal efficacy of plasma in artificial saliva. Rather, the direct exposure of bacteria to plasma or plasma-generated gas throughout treatment may be important for bacterial killing. Although plasma-treated solutions can also inactivate bacteria^34, 35, 42^, direct contact between plasma and bacteria more rapidly sterilizes than does the indirect method^43^. This difference in efficacy may be related to reactive species with very short half-lives or free electron accumulation within plasma being crucial for bacterial inactivation. Further studies are needed to examine these possibilities.

## Conclusion

In this study, microwave-pulsed NTAPP was used to control bacterial contamination in artificial saliva, and plasma effects on gingival tissue cells were identified. The results suggest that antibacterial activity of microwave-pulsed NTAPP can be enhanced in artificial saliva with inflammatory response suppression in gingival cells. Although further investigations are required *in vivo*, our results suggest that microwave-pulsed NTAPP may be a useful alternative tool in clinical treatment of periodontal diseases because of its dual effects of bacterial inactivation and suppression of cellular inflammation.

## Materials and Methods

### Microwave-pulsed non-thermal atmospheric pressure plasma and sample treatment

Microwave-pulsed NTAPP was used in the study. The plasma impedance system consisted of a solid-state power oscillator, circulator, directional coupler, three-stub tuner, gas-flow meter, and plasma generator (Fig. 1a). A radio frequency signal generator (Agilent, Santa Clara, CA, USA) was connected to the resonators using an HY 3005D-3 DC power supply (Mastech Power Supply, CA, USA), which used a frequency of 930 MHz and a power of 40 W. Ar was used as the feeding gas, with a degree of plasma ionization of 10^13^/cm^3^ and electron temperature of 1.2 eV^44^.

Because extended plasma discharge produces heat, we determined optimal treatment conditions for antibacterial and cellular activities without thermal effect. The distance between the end of the plasma plume or gas flow and upper surface of the solution containing bacteria or cells was set at 10 mm (Fig. 1c). Feeding gas, frequency, and power were maintained as described above. Samples treated with Ar gas only were used as controls. Ions and radicals generated by plasma were characterized using an optical emission spectroscope comprised of a collimator lens, optical fiber-core (diameter, 600 µm), and spectrometer.

### Antibacterial activity test

Two to three *S*. *aureus* colonies were suspended in 1.8 ml Luria-Bertani (LB) liquid and then 10 µl of this suspension was inoculated into 15 ml of new LB liquid. After incubation at 37 °C for 16 h with shaking at 140 rpm, bacteria were washed with saline (0.85% NaCl solution) once and re-suspended in new saline. Then, the bacterial suspension was diluted to reach a 1.0 absorbance at 600 nm wavelength with 10^6^ bacterial cells per ml. The diluted bacterial suspension was divided into two conical tubes (20 ml per tube) and centrifuged at 3134 × g for 5 min. After discarding the liquid, the bacterial pellet was re-suspended in 20 ml of saline or artificial saliva. The artificial saliva was prepared following the Fusayama-Meyer method^45^. The chemical composition of the artificial saliva is shown in Table 1. Supplementary Table S2 illustrates the composition of artificial saliva solutions after omission of individual chemicals. Aliquots of 1 ml of bacterial suspension (10^6^ cells) in saline or artificial saliva were placed on Petri dishes and exposed to plasma or Ar gas (control) for 1 min, 3 min, 5 min, or 7 min. After plasma or Ar gas exposure, the bacterial suspension was transferred to microtubes and incubated at room temperature for 0 min or 30 min. Next, the bacterial suspensions were serially diluted and 100 µl of the diluted suspension was spread onto LB agar plates. Plates were incubated at 37 °C overnight; CFU were counted the next day. All experiments were conducted three times. The same procedure was applied to bacteria treated with plasma in artificial saliva that had individual chemicals omitted.

**Table 1:** Components of saline and artificial saliva.

Solution	Components	Concentration (mM)
**Saline**	NaCl	145.0
Artificial saliva	NaCl	6.8
	KCl	5.4
	CaCl_2_ · 2H_2_O	5.4
	NaH_2_PO_4_ · 2H_2_O	5.0
	Na_2_S · 9H_2_O	0.021
	Urea	16.5

### Scanning electron microscopy

Bacterial cell morphology after plasma treatment was determined using a field-emission scanning electron microscope (JEOL 7100F, Tokyo, Japan). Bacterial cells in artificial saliva were treated with plasma or Ar gas (control) for 5 min, as described previously. Next, bacterial cells were washed with saline three times and then processed further for scanning electron microscopy (SEM) analysis, following previously described procedures^46^. Platinum-coated samples were used for SEM at 5 kV and 50,000× magnification.

### Temperature, pH, and reactive species analyses

Solution pH and temperature were measured with a portable pH meter (Oakton Instruments, Vernon Hills, Il, USA) and an infrared thermometer (Mini Infrared Thermometer Model SK-8700, SATO Keiyoki Co., Ltd, Tokyo, Japan) immediately following exposure to plasma for 1 min or 5 min. Untreated distilled water was used as a reference control. We also assessed ROS and reactive nitrogen species in plasma-treated solutions by measuring OH and NO levels, respectively. Terephthalic acid (TA, Sigma Aldrich, Winooski, VT, USA) was dissolved in saline or artificial saliva (20 mM concentration) and then the solutions were exposed to plasma or Ar gas (control) for 1 min or 5 min. Fluorescence emission level by hydroxy-TA was measured at 310_ex_/425_em_ nm with a multi-detection microplate reader (BioTek, Winooski, VT, USA). To detect NO, 4-Amino-5-Methylamino-2′,7′-Difluorofluorescein (DAF-FM, Life Technologies, Carlsbad, CA, USA) was used. After treatment with plasma or Ar gas, DAF-FM was added immediately to the solutions to achieve a final concentration of 1 µM. Lastly, fluorescence was monitored at 495_ex_/515_em_ nm using a multi-detection microplate reader.

### Cellular response analysis

HGFs (CRL-2014, ATCC, Manassas, VA, USA) were obtained between passages three and seven for treatment with gas only or plasma, using the same conditions used in the antibacterial activity experiment. Cell suspensions of 1 ml (1 × 10^4^ cells) were placed in each well of a 24-well plate and then treated with plasma for 1 min, 3 min, or 5 min. After treatment, HGFs viability was determined using the EZ-CyTox WST assay (Daeillab, Seoul, Korea) following the manufacturer’s instructions.

The influence of plasma treatment on inflammatory cytokine secretion was determined by examining TNF-α release from cells. HGFs (1 × 10^4^ cells) treated with or without plasma were stimulated with LPS (Sigma-Aldrich, St. Louis, MO, USA) to induce inflammation. Based on the results, 1.0 µg/ml of LPS concentration was chosen to induce inflammation. WST assays were performed to determine cell viability after 24 h. Untreated cells [con (−)] and LPS-treated cells without plasma [LPS only] were used as controls. The experimental groups included untreated normal cells designated as con (−), LPS-stimulated cells, and plasma-treated, LPS-stimulated cells. TNF-α concentrations in culture supernatants were measured by enzyme-linked immunosorbent assay (BioVision, Inc., Milpitas, CA, USA).

### Statistical analysis

Quantitative data are reported as means ± standard deviations. Because the sample number in each group was small (3 to 9), the Mann-Whitney U test was used to compare groups^47^. Statistical significance was determined by a *P* value less than 0.05.

## Electronic supplementary material

Supplementary Table S1

Supplementary Table S2
