# Supplementary material for: Antibacterial activity and effect on gingival cells of microwave-pulsed non-thermal atmospheric pressure plasma in artificial saliva
Source: Sci Rep. 2017 Aug 21;7:8395. doi: 10.1038/s41598-017-08725-0 (PMC5567204; doi:10.1038/s41598-017-08725-0)
Supplement: Supplementary file 2 — Supplementary Table S2 [file 41598_2017_8725_MOESM2_ESM.pdf]

Supplementary Information

Antibacterial activity and effect on gingival cells of microwave-pulsed non-thermal atmospheric pressure plasma in artificial saliva

Sang-Hee Seo[1,2], Ihn Han[3], Han Seol Lee[3], Jin Joo Choi[3], Eun Ha Choi[3], Kyoung-Nam Kim[1,2] , Gyungsoon Park[3], Kwang-Mahn Kim[1,2]

[1] Department and Research Institute of Dental Biomaterials and Bioengineering, Yonsei University College of Dentistry, Seoul, 03722, Korea

[2] BK21 PLUS Project, Yonsei University College of Dentistry, Seoul, 03722, Korea

[3] Plasma Bioscience Research Center, Kwangwoon University, Seoul, 01897, Korea

Supplementary Table S2. Modified artificial saliva solutions with individual components omitted

Omitted component	Components	Concentration (mM)
NaCl	KCl	5.4
	$CaCl_2 \cdot 2H_2O$	5.4
	$NaH_2PO_4 \cdot 2H_2O$	5.0
	$Na_2S \cdot 9H_2O$	0.021
	Urea	16.5
KCl	NaCl	6.8
	$CaCl_2 \cdot 2H_2O$	5.4
	$NaH_2PO_4 \cdot 2H_2O$	5.0
	$Na_2S \cdot 9H_2O$	0.021
	Urea	16.5
$CaCl_2 \cdot 2H_2O$	NaCl	6.8
	KCl	5.4
	$NaH_2PO_4 \cdot 2H_2O$	5.0
	$Na_2S \cdot 9H_2O$	0.021
	Urea	16.5
$NaH_2PO_4 \cdot 2H_2O$	NaCl	6.8
	KCl	5.4
	$CaCl_2 \cdot 2H_2O$	5.4
	$Na_2S \cdot 9H_2O$	5.0
	Urea	16.5
$Na_2S \cdot 9H_2O$	NaCl	6.8
	KCl	5.4
	$CaCl_2 \cdot 2H_2O$	5.4
	$NaH_2PO_4 \cdot 2H_2O$	5.0
	Urea	16.5
Urea	NaCl	6.8
	KCl	5.4
	$CaCl_2 \cdot 2H_2O$	5.4
	$NaH_2PO_4 \cdot 2H_2O$	5.0
	$Na_2S \cdot 9H_2O$	0.021
